# Chemometric Evaluation of THz Spectral Similarity for the Selection of Early Drug Candidates

**DOI:** 10.1038/s41598-017-14819-6

**Published:** 2017-11-06

**Authors:** Lukasz A. Sterczewski, Kacper Nowak, Boguslaw Szlachetko, Michal P. Grzelczak, Berenika Szczesniak-Siega, Stanislawa Plinska, Wieslaw Malinka, Edward F. Plinski

**Affiliations:** 1 Wroclaw University of Science and Technology, Faculty of Electronics, 50-370 Wroclaw, Poland; 2 Wroclaw Medical University, Department of Chemistry of Drugs, 50-556 Wroclaw, Poland; 3 Wroclaw Medical University, Department of Inorganic Chemistry, 50-556 Wroclaw, Poland; 4 Princeton University, Department of Electrical Engineering, Princeton, New Jersey 08544 USA

## Abstract

In this paper we discuss the link between the domain of physical parameters – molecular descriptors of a drug, and terahertz (THz) spectra. We measured the derivatives of the well-known anti-inflammatory drug Piroxicam using THz spectroscopy and employed Principal Component Analysis to build similarity maps in the molecular descriptor and spectral domains. We observed, that the spatial neighborhood on the molecular descriptors map is highly correlated with the spectral neighbourhood within a group of structurally-similar molecules. We built a Partial Least Squares (PLS) predictive model to quantify the relationship between the spectra and the melting point, which can guide the selection of early drug candidates.

## Introduction

Drug discovery is a costly, complex, and time-consuming process. In recent decades much attention has been devoted to the development of methods aiming to support the choice of new candidates for pharmaceuticals with fewer side-effects, lower toxicity, and higher efficacy. For instance, initial selection of promising drug candidates used to be governed by the well-known Lipinski’s rule of five (Ro5)^1^. In order to exhibit biological or pharmacological activity, the molecular mass *M*_*r*_ of an orally active substance should be below 500 g/mol, its octanol-water partition coefficient (log*P*) should not exceed 5, the candidate is expected to have no more than 5 hydrogen bond donors (HBD), and no more than 10 hydrogen bond acceptors (HBA). Notably, each parameter, known as a molecular descriptor, is constrained to be within a proper range of values – multiples of five.

Recently, this approach has been criticized^2^. The authors pointed out, that there are many exceptions from the Ro5 and proposed a numerical quantity to characterize the *chemical beauty of drugs*. In contrast to the Ro5, the obtained evaluation is no longer binary (accept/reject) but provides a continuous scale spanning between 0 and 1, useful for comparative analysis. The newly proposed evaluation method (QED – quantitative estimate of drug-likeness) is based on the histograms of molecular descriptors of the marketed drugs, and assumes that a drug-like candidate matches the statistical pattern – desirability functions obtained from empirical distributions of the descriptors’ occurrence. A good match with the model yields high scores in the chemical “beauty contest”, corresponding to potential pharmaceutical activity.

The above selection technique, together with its numerous variants was made possible by the rapid increase in computational power, giving birth to a new class of methods, known as *in silico* drug design. For instance, information about the relationship between the chemical structure and pharmacological activity^3^ (SAR – structure-activity relationship) obtained from large databases is conveniently employed at the drug design stage to predict its bioactivity and toxicity. The search for chemical structures is guided by an intuitive concept of similarity: molecules with a similar structure to those pharmaceutically-active, but with intentionally engineered physical properties, can potentially mimic or even supersede them. Consequently, the exploration of patterns in pharmaceutical data that yield potential activity is of large interest.

If we carefully analyze the importance of aforementioned molecular descriptors in drug research, and realize that most of them correspond to physical properties (solubility, melting point, molecular mass etc.), an idea to employ spectroscopy in the selection of drug candidates may arise. Particulary in the terahertz (THz) region, where the vibrations of the crystal lattice of many solid state drugs at room temperature can be observed^4^. These vibrations correspond to the domain of physics rather than chemistry. Regrettably, an explicit link between the spectrum and the physical parameters may not be reasonably justified. Furthermore, unlike in the near- and mid-infrared, far infrared spectra of solids do not have a straightforward way of interpretation. They provide a lot of information but seem to be too complex to analyze and resolve. Despite the fact that molecular modeling (*i.e*. involving DFT – Density Functional Theory) can explain the origin of THz absorption peaks, it usually involves supplementary X-ray diffractometry (XRD) studies^5^, making analytical studies in the far infrared laborious and costly. Instead, one can utilize THz spectroscopy as a tool to detect physical (crystal lattice) similarity because the dynamics of the structure is reflected in the THz spectrum. For simplicity, we can assume that the origin of the spectral peaks is not well known but some information about the mutual similarity within a group of structurally-similar solid drug candidates is encoded in the spectra. Then, thanks to chemometric methodology that employs mathematics and computer science to simplify problems in chemistry and biology^6^, we can project the high-dimensional spectral and molecular descriptor data to two dimensions, quantify mutual similarities mathematically, and finally try to correlate the two domains. In other words, we can consider the THz spectra as data vectors representing some unknown features, which treated algorithmically generate complementary maps with characteristic clusters.

Among many chemometric techniques applicable here, two algorithms deserve special attention: Principal Component Analysis (PCA) and Partial Least Squares (PLS)^7^. As opposed to many machine learning techniques, these rely on a simple matrix decomposition and data compression rather than on an artificial neural structure and its iterative training. In particular, PCA, thanks to its strong deconvolution capabilities^8^, has already found applications in resolving ternary mixtures of THz spectral compounds^9^, classification of drugs-of-abuse mixtures with diluents^10^, studies on protein-antibody interactions^11^, and materials classification^12^. Regarding the similarity studies in the THz, those were limited mainly to isomers^13^ and polymorphs^14^ – structurally identical compounds different in crystal structure, but did not include molecules sharing the same scaffold with different substituents. Consequently, the application of chemometric techniques utilizing the THz spectra to support the well established drug design routine is yet to be evaluated.

Still, a question may arise: would it make sense to use THz spectra for drug candidate selection, if the chemical structure similarity does not correspond to the THz spectral similarity^15^? Firstly, THz waves have already proven its usefulness in numerous pharmaceutical applications^16–19^ by revealing changes in the crystal lattice that cause varying bio-availability. Secondly, in our previous work, which is the basis of this paper, we have identified a relationship between the similarity of selected physical parameters, and the similarity of THz spectra within a group of structurally-similar molecules^20^. We propose a method described in this paper to predict some of those physicochemical parameters using the THz spectra, which can act as a guide in the selection of early drug candidates. For instance, passive intestinal drug absorption of poorly soluble drugs is, in general, higher for those with a lower melting point^21^.

Undoubtedly, it is an overstretch to say that drug activity is encoded in the THz spectrum and molecular descriptors in a straightforward way due to the immense complexity of drug action mechanisms. Nonetheless, if one follows the standard procedure of pharmacophore selection (groupings with known pharmacological activity), followed by an optimization of molecular properties, the use of physical similarity detected by means of THz spectroscopy of candidates selected in such a process may also help to predict the activity of the untested drugs based on the distribution of those with confirmed activity.

**Figure 1:**
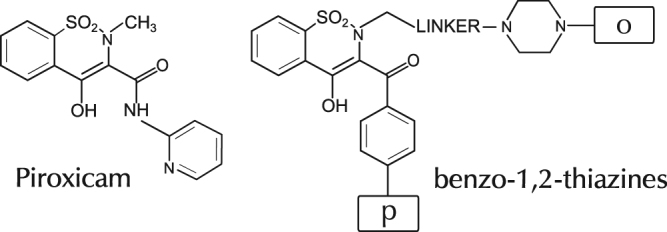
Structural formula of Piroxicam and investigated analogues – benzo-1,2-thiazines. Letters ‘p’ and ‘o’ indicate the position wherein different fragment structures were substituted.

To demonstrate the proof-of-concept, we have analyzed 27 structurally-similar analogues of the well-known non-steroidal anti-inflammatory drug – Piroxicam, proven to be well tolerated in animal model in contrast to the reference drug^22–24^. These analogues – benzo-1,2-thiazines (see Fig. 1) – share the same scaffold (core part) of the base drug but have different substituents denoted by letters ‘p’ and ‘o’, and natural numbers, *i.e*. ‘1p’, ‘2p’, ‘1o’, ‘2o’ etc., where substituents sharing the same both number and letter were identical (for a detailed list of the samples please see Table S1 in the Supplementary Information). The letters ‘p’ and ‘o’ used in the sample name correspond to Polish words that describe the base of the molecule (lower fragment structure) – *podstawa*, and the molecular tail – *ogon*, which are kept untranslated for consistency with the reference work^25^. Within our dataset, we studied one substance in two polymorphic states: sample 2a shares the same structural formula as 2b and 66, but has a different crystal structure. Elemental analysis (C, H, N), chromatography (TLC), and spectral studies (FT-IR/IR, ^1^H NMR, MS) confirmed the purity^†^ of the investigated compounds after synthesis, and can be found herein^25^

1

 The samples in a crystalline form were first ground into fine particles using a pestle and mortar, next mixed with a spectroscopic grade polyethylene (PE) matrix (10% of drug with one exception of 20%), and finally compressed into 400 mg pellets with a diameter of 13 mm, and thickness of approximately 3 mm. Please see Methods for more details regarding the sample preparation. In the spectroscopic experiment, the samples were inserted into a rotary sample holder, and probed sequentially by a THz beam, which allowed us to retrieve their optical properties^26^.

Having acquired THz spectroscopic data, we performed a two-stage analysis that enabled us to discover that the THz spectral neighborhood reflects the molecular descriptors’ similarity. In the first step, we measured the mutual similarity of the samples based on the molecular descriptors set provided *a priori* and in the next, we used the THz spectroscopic data acquired in the *a posteriori* experiment. In each phase we employed principal component analysis (PCA), which is frequently applied to visualize the mutual similarity of high-dimension data, *i.e*. spectra, by projecting the data onto a 2D map. Spatial neighborhood on such maps defines our analogy criterion^27^. A brief but intuitive explanation of the PCA method is provided in the Supplementary Information. Additionally, for the spectroscopic data, we harnessed the agglomerative hierarchical cluster tree to visualize how the position of absorption peaks contributes to the formation of clusters of similar molecules. In the final step, we try to link the *a priori* and *a posteriori* generated maps and utilize their similarity to predict selected physicochemical parameters.

## Results

### A priori analysis

In *a priori* analysis, we studied the similarity of the physical parameters before the spectroscopic measurements were taken. In addition to the aforementioned Lipinski’s rule of five (Ro5) descriptors (melting point MP, polar surface area PSA, octanol-water partition coefficient log*P*, number of hydrogen donors HBD and acceptors HBA, and molecular mass *M*_*r*_), we extended the number of variables by introducing the QED descriptors:^2^ number of rotable bonds (ROTB) and aromatic rings in the structural formula (AROMS). Based on these selected parameters, a prediction of dermal^28^ or oral bioavailability^29^ has been demonstrated.

**Figure 2:**
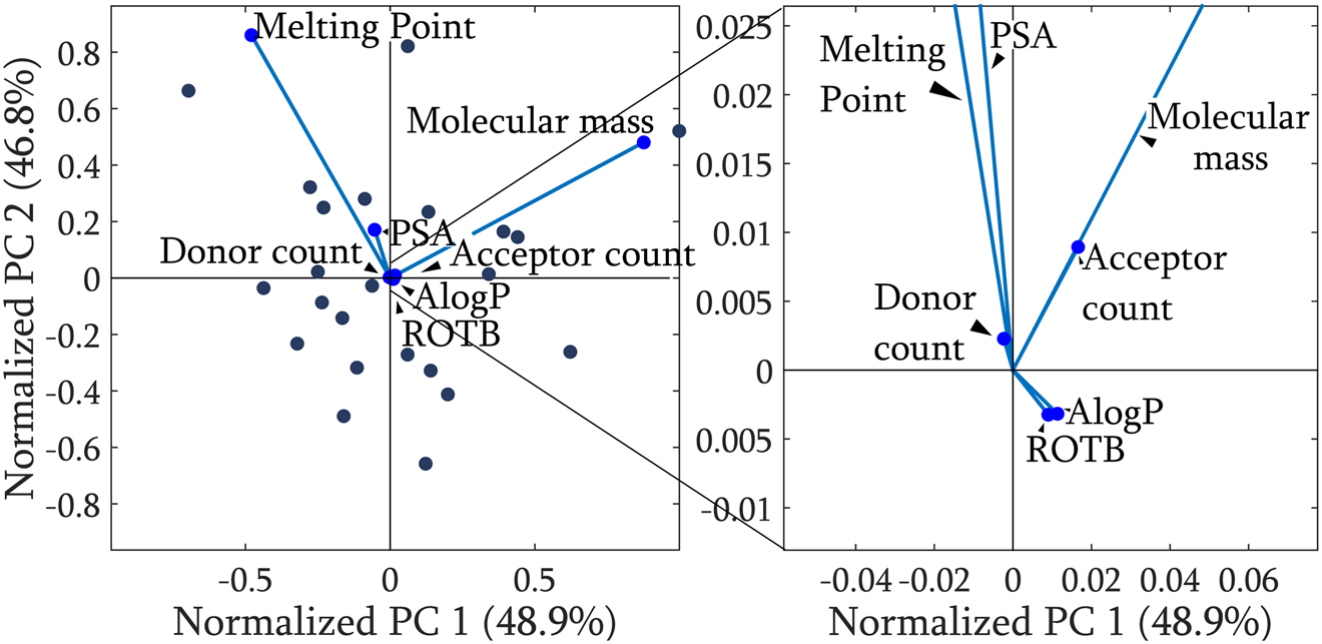
Biplot showing the significance of each descriptor (blue line vector) projected onto 2D space. The melting point (MP) and molecular mass *M*_*r*_ are sufficient to explain more than 95% of the data variance in *a priori* analysis.

We projected the molecular descriptors data onto 2D space. The results are shown in a biplot in Fig. 2, which visualizes the significance of each descriptor (blue vector lines) in the principal component space with the sample data points marked as dots. We clearly see the two dominant vectors: the melting point and molecular mass, which are orthogonal in 2D space. The contribution of the remaining descriptors is residual (<5%), thus the position of the samples in the PCA plot depends only on these two parameters. The biplot also shows that the polar surface area and melting point are correlated because they point in nearly the same direction. Indeed, the correlation coefficient of these two vectors within the dataset was equal to 0.464, as opposed to the correlation of the dominant vectors equal to only 0.025.

**Figure 3:**
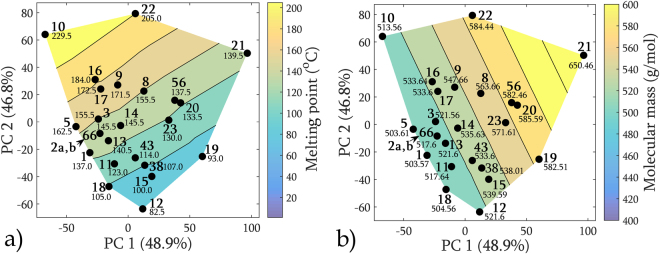
PCA score map coupled with a contour plot of the numerical values of two dominant molecular descriptors. The growth direction follows that pointed by the dominant vectors in the biplot. The sample number is written in bold. Sample 19 has not been measured spectroscopically.

If we couple the direction pointed by the two dominant descriptors – melting point and molecular mass – with a contour plot of their numerical values, as in Fig. 3, we can see that their growth direction follows that pointed by the vectors. The melting point values rise diagonally from the South-East to North-West, whereas the molecular mass follows the orthogonal direction. Such a map was used in the further part of our consideration to characterize mutual similarity and link the descriptor’s domain with the spectroscopic domain.

### A posteriori analysis

The *a posteriori* analysis was conceptually similar to the previous one but the subject of our considerations were spectroscopic data. In raw form (time domain traces) they were not suitable for similarity studies, therefore we calculated their frequency spectra followed by removing the response of the diluting medium, and normalizing the absorption spectra using the standard normal variate (SNV) to ensure an equal contribution into the statistical model. The signal processing steps, including a comparison of different normalization techniques, as well as their influence on the shape of the PCA maps can be found in the Supplementary Information.

### Visualization of spectral similarity using a dendrogram

**Figure 4:**
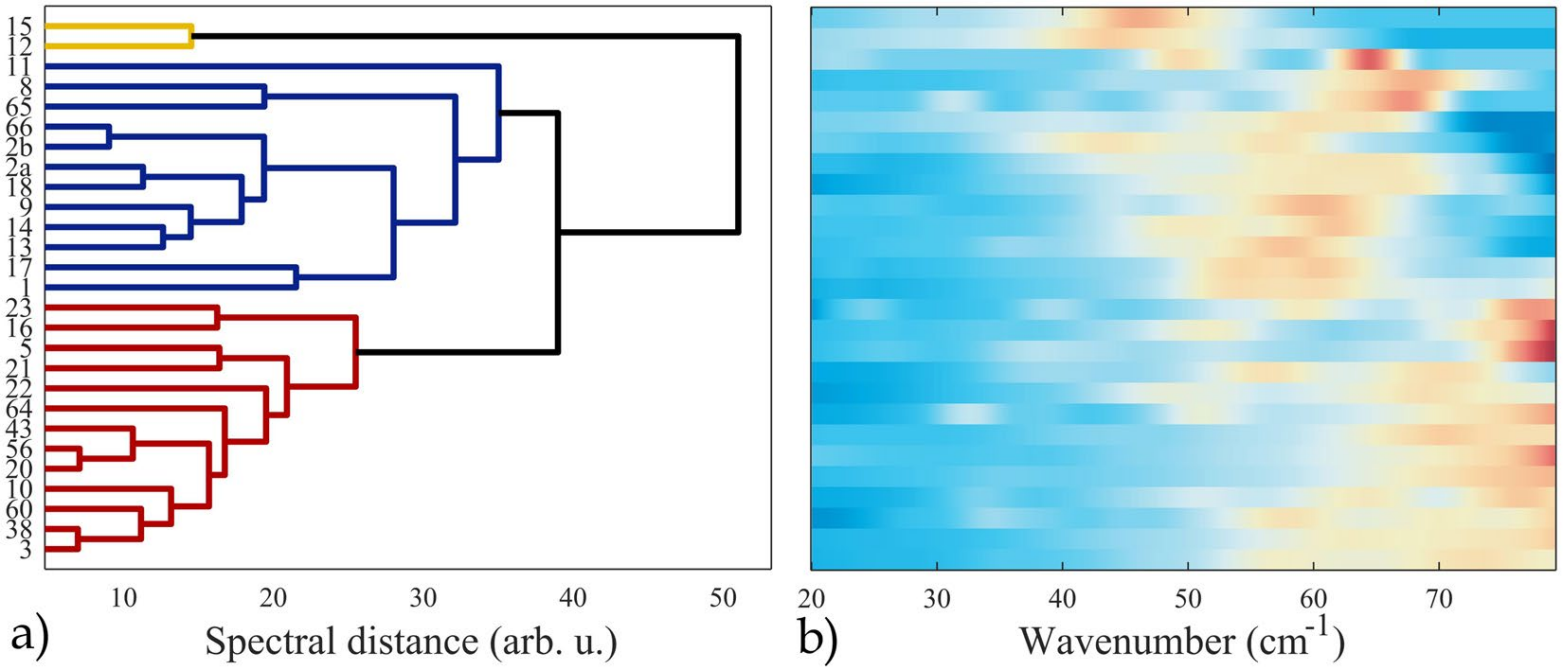
Dendrogram chart **(a)** representing the hierarchical structure of mutual similarity measured as the Euclidean distance between the spectra and the corresponding spectral intensity image **(b)**. Notably, three spectral groups are distinguishable: top – with the peaks located in the center, middle – peaks around 50–60 cm^−1^, and bottom – around 70 cm^−1^.

A hierarchical cluster tree, which measures the point-wise similarity of the THz spectra, is shown in Fig. 4 together with a spectral intensity image. Although the spectral similarity can be quantified in many ways, we calculated a simple point-wise Euclidean distance of high-dimensional spectral vectors. Intuitively, the lower the Euclidean (spectral) distance, the more similar the spectra are. By means of the hierarchical clusterization, which used the UPGMA (Unweighted Pair Group Method with Arithmetic Mean) algorithm to build the tree, nearest spectra are initially grouped into mini-clusters. In further steps, the mean distance between the pairs of such clusters is used as the similarity criterion to link them into larger clusters. As a result, the algorithm builds a hierarchical structure with an increasing spectral distance until all clusters meet in the root. The resulting tree enables to classify the investigated samples into groups with an arbitrary level of similarity.

A spectral intensity image coupled with the dendrogram plot illustrates the usefulness of the clusterization. At a spectral distance of approximately 38 units, three distinct groups of spectra with different absorption peak positions can be identified: the top with peaks in the center, the middle with peaks around 50–60 cm^−1^, and the bottom with peaks in the high-wavenumber region. Two neighboring spectra on the intensity map and the dendrogram are even more similar, for instance the two bottom samples: 38 and 3, which have only two wide absorption peaks above 50 cm^−1^. Clearly, the dendrogram has an easy-to-follow physical interpretation. Most importantly, it illustrates that samples with the same fragment structures do not have to be spectrally similar in the THz domain, despite their chemical similarity. This is because they can have different crystal structures or hydrogen bond interactions, which are responsible for the THz absorption features. Importantly, this confirms the previously referenced observations^15^.

### PCA on THz spectroscopic data

**Figure 5:**
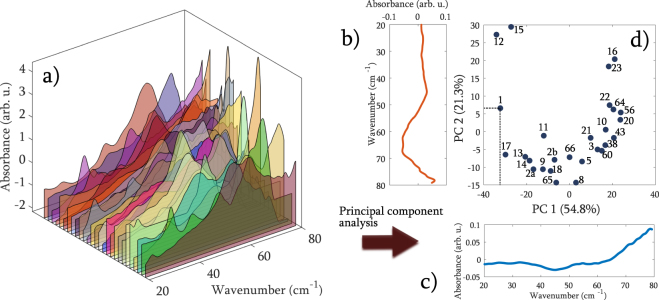
Representation of the SNV-normalized THz spectra **(a)** in a new coordinate system to visualize similarity. The eigenvectors **(b)** and **(c)** are multiplied by the corresponding coordinates of the PC scores as shown with dashed lines for Sample 1 in **(d)** to reconstruct the original spectroscopic data (without the mean).

For the visualization of the mutual similarity of the THz spectra on a 2D plot, we reduced the dimension of the input data from 1137 down to two using PCA, preserving 76.1% of the data variance. The PCA algorithm represents the input spectra (Fig. 5a) in a new coordinate system, namely the PC loadings, shown in Fig. 5b and c. These basic vectors “mixed” in the proportion provided by the PCA scores can be used to reconstruct the original spectrum. A plot of the “mixing coefficents” – PC scores one against the other, as in Fig. 5d was used for similarity studies: samples in close neighborhood are mutually similar.

### Linking the a priori and a posteriori maps

**Figure 6:**
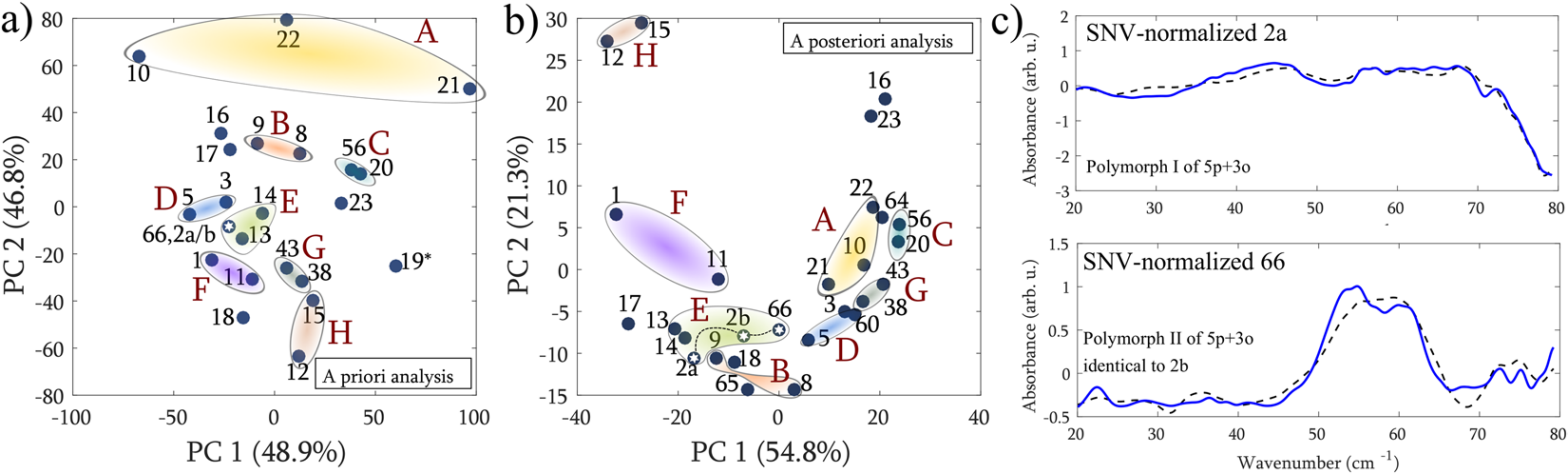
**(a,b)** Linking the a priori (non-spectral) and a posteriori (including THz spectra) generated PCA maps. Eight groups A–H are distinguished, which share the same properties in the spectral domain. The sample number is written in bold. **(c)** Structurally identical samples but existing in two polymorphic forms. The non-compressed spectra (solid lines) are shown with their reconstruction from five PC loadings (dashed lines).

The *a posteriori* generated PCA map after SNV normalization (Fig. 5d) was used in comparative studies with the *a priori* generated one (Fig. 3). An attempt was made to link the two maps, as shown in Fig. 6. It was discovered that samples lying in a close neighborhood on the molecular descriptors map share the same property in the spectral domain. Groups of the corresponding samples are encircled and marked with subsequent letters of the alphabet (A–H). There are a few exceptions but in general ~85% of the full analogues (containing both ‘p’ and ‘o’ substituents) obey this rule, hence we can conclude that there is a link between the physical domain of molecular descriptors, and the THz spectral domain. On the other hand, PCA assumes a possibly oversimplified linear dependence between the variables, which may be insufficient to explain the sophisticated relationship between these two domains. Instead, a non-linear dimensionality reduction technique that employs a manifold to represent the data can be used.

Additionally, in the *a posteriori* map (Fig. 6b) three samples of a structurally identical compound, but in two different polymorphic forms: 2a (polymorph I), 2b (polymorph II), and 66 (polymorph II, 20% concentration) lie in a close neighborhood. For better visibility we marked them with stars and connected them with dashed line. This feature of our method may be useful in polymorphic studies, also. Notably, a visual comparison of the spectra (Fig. 6c) would not yield a conclusion of their relationship. Although there is no visual similarity between these spectra, their neighborhood was detected by the PCA algorithm.

### Physical parameters coupled with the PCA score maps

**Figure 7:**
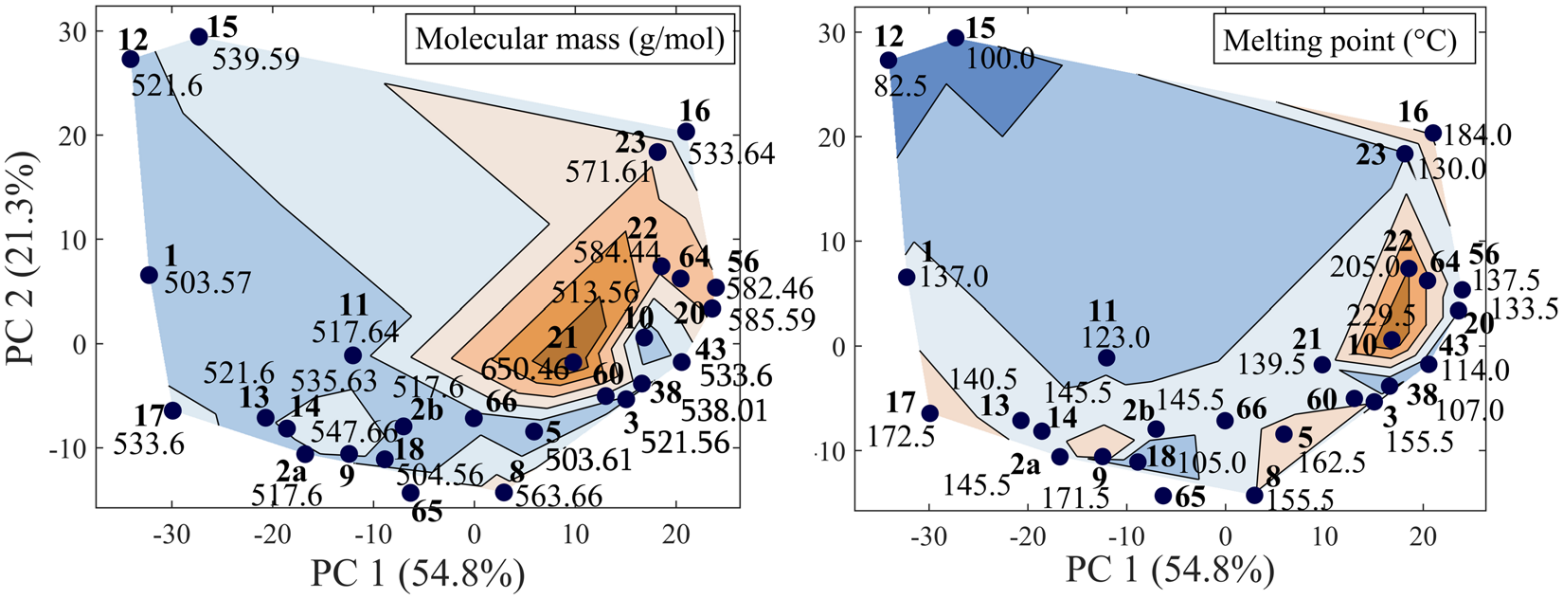
Representation of different physical parameters coupled with a PCA score map: molecular mass (left), melting point (right). The sample number is written in bold.

Similarity detected by comparing the *a priori* and *a posteriori* maps is also reflected in the PCA score maps coupled with the numerical values of the molecular descriptors shown in Fig. 7. In selected regions of the maps, samples with similar numerical values tend to agglomerate. Such behavior was observed only for the two dominant descriptors: molecular mass *M*_*r*_ and melting point MP. The maps may be useful to determine the possible positions of the sample on the spectral map if one carefully performs the *a priori* analysis first.

### An attempt to predict a physical parameter through PLS regression

We employed the partial least squares (PLS) regression algorithm to predict the melting point from a THz spectrum, thus making an attempt to link the molecular descriptors and THz spectral domain using one more technique (for a brief introduction, please see the Supplementary Information). We have performed five thousand prediction experiments, given in two variants: four samples left out, and eight samples left out. In each iteration we chose a random test group, averaged the prediction results, and calculated the estimation error. The selected number of PC components in the PLS model allowed to achieve approximately 10% of the mean relative error of the melting point prediction, when validated with the training set. This precluded the over-training of the algorithm, thereby enabling it to estimate the parameters of new, unknown sample spectra.

**Figure 8:**
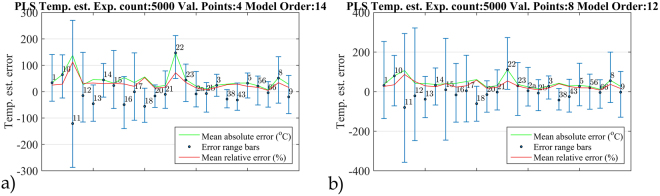
PLS melting point prediction in the four-left-out **(a)** and eight-left-out **(b)** test together with the mean absolute and mean relative error.

Figure 8a and b show the results of the four-left-out and eight-left-out experiments, respectively, together with the mean absolute error $\hat{e}$ and the mean relative error ${\hat{e}}_{R}$. Additionally, the error bars (with maximum *e*_*α*_ and minimum *e*_*ω*_ values of prediction errors) are presented to evaluate the estimation performance. We can see that the mean absolute error of the melting point prediction of selected substances exceeds the true value of this parameter. Moreover, the mean relative error in the range of 10–50% is too high to assume any significant relationship between the measured THz spectra and the tested parameter for these samples using PLS. This can be attributed to the oversimplified linear model, as in the PCA case, which may not be sufficient to explain the sophisticated spectral-physical parameter dependencies. Obviously, the lack of a valid model, which requires a large training database also contributed to the unsatisfactory results.

Despite the fact that we observed a reasonably good prediction capability for some analogues using this simple method, the selection of the training set considerably affects the melting point estimation error. When the proper training sets are used, the relative error of the prediction is lower than 30%, whereas in the wrong case, the relative error of the prediction is greater than 70%. There is one member of the wrong training sets – sample 10 (1p + 22o), whose presence leads to large prediction errors and it can be interpreted that this particular substance is somehow different from the others. Indeed, it is the only one within our set that has a rigid joint between the Piroxicam scaffold and its substituent. The problems with large prediction errors can be potentially mitigated by building a neural network model or using more flexible nonlinear prediction tools. Additionally, the methodology of choosing the training sets to minimize the prediction error needs further research.

### Reproducibility

A key element in similar studies is reproducibility. While the *a priori* analysis involves the characterization of physicochemical parameters, which relies on well-established molecular models or simple measurements of single quantity, such as temperature, results of multi-band spectroscopic studies as part of *a posteriori* analysis are known to depend upon many factors that introduce variation in results.

Firstly, the THz system exhibits a slight drift of parameters over time, which we characterize by performing the reference measurement of the pure PE sample twice: before and after all drug samples are measured. The standard deviation of the absorbance calculated by referencing the two measurements taken within 40 minutes was 1.2⋅10^−2^. It translates into approximately 5% of mean error in absorbance for the weakest absorbers, and less than the 1% for the strongest ones, when 25 signals are coherently averaged.

**Figure 9:**
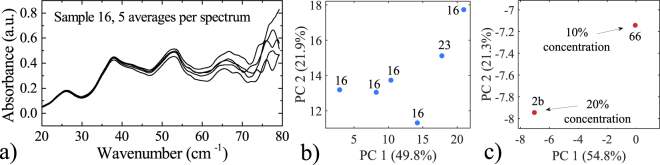
Reproducibility of the spectroscopic data projected onto the PCA map. (**a**) Coherently averaged THz spectra obtained from 5 scans for each plotted together to evaluate the effect of noise at different frequencies. **(b)** Data from (**a**) projected onto the PCA map. The variance explained by the axes in this demonstration has slightly changed due to the introduction of the new data points. **(c)** PCA map showing the neighborhood of the same compound in different concentrations (magnified version of Fig. 6b).

Secondly, the signal-to-noise ratio of the THz spectrum varies with frequency, causing higher frequency-data to differ from scan to scan more significantly than at lower frequencies. To illustrate this effect, we plotted the THz spectra obtained by averaging 5 scans acquired at a constant position of the rotary sample holder (Fig. 9a) of moderately absorbing Sample 16. Up to 50 cm^−1^ the agreement between the spectra is excellent with the relative standard deviation lower than 5%. It increases up to 30% at 80 cm^−1^, however the increased number of averages in an acquisition *n* should scale this number by the square root of *n*, down to less than 15% for 25 averages. The reason of those fluctuations can be attributed to the change in optical alignment of the free-space THz antennas, and the irreproducibility of the sample position after a full rotation of the sample holder is made.

To quantify the effect of varying intensity of THz spectra on the position on the PCA maps, we projected the spectra from Fig. 9a, as shown in Fig. 9b. The distance from the center point is approximately 10 units along the PC1 axis, and 4 units along the PC2 axis. Compared to their span, it translates into 14%, and 10% in the horizontal and vertical direction, respectively.

Finally, we perform an analysis of pellet-to-pellet variation and its effect on the change of the position on the PCA map. We measured one compound in two different concentrations (10 and 20%) – pellets denoted as 66 and 2b. The projection of the two samples onto the PCA map is shown in Fig. 9c, which is a magnified version of Fig. 6b. The two samples have been correctly detected as neighbors, shifted by 10% in the horizontal, and 2% in the vertical direction defined by the PC1 and PC2 axes, respectively.

## Discussion

In this work, we investigated the relationship between the THz spectra of structurally-similar drug candidates – Piroxicam derivatives and their physicochemical parameters. We employed two well-known chemometric tools in our methodology: principal component analysis (PCA) and partial least squares regression (PLS). Using PCA, we projected the provided *a priori* molecular descriptors based on the Lipinski’s Rule of Five and the quantitative estimate of drug-likeliness (QED) onto 2D space, detecting that only two variables were responsible for >95% of the data variance: the melting point and molecular mass. Next, we applied the PCA method to the acquired THz spectra in the *a posteriori* analysis, reducing the input spectral vector from 1137 down to two dimensions, while preserving 76.1% of the variance. We generated a THz spectral similarity map by plotting the principal component scores of the first two components.

We discovered that the neighborhood on the molecular descriptors map, determined dominantly by the melting point and molecular mass coordinates, corresponded to the neighborhood in the THz spectral domain. Approximately 85% of the full analogues (containing both ‘p’ and ‘o’ substituents) obey this rule, indicating that there might be a link between the molecular descriptors and THz spectral domains. Notably, the degree of correlation depends on the normalization method. In our approach, the Standard Normal Variate (SNV) method was applied, which amplifies the weaker THz peaks and thus enables the discovery of the aforementioned interesting relation.

The THz spectra in our similarity analysis were also clusterized using the dendrogram – a chart that represents the hierarchy of Euclidean distances between the spectra understood as vectors. Visualizations obtained with the dendrogram confirmed the results of clusterization created with PCA analysis – showed that structurally similar compounds seem not to be spectrally similar in the THz.

In the last step, we made an attempt to predict the melting point of our benzo-1,2-thiazines from the THz spectrum using PLS. If a good training set is chosen, the relative error of the melting point prediction goes below 30%, which can be considered a satisfactory result. Consequently, in our opinion the errors are low enough to anticipate such parameters based on THz spectra, if the conditions of structural similarity of analogous compounds are met. It is worth noting, that the methodology of choosing the training sets in order to minimize the prediction error needs further research.

We believe that an initial selection of promising candidates can be supported by means of THz spectroscopy and chemometrics. Thanks to the relationship between the molecular descriptors domain and THz spectra within a group of structurally-similar compounds, spectroscopic measurements can potentially be used as a guide in early drug development and discovery to promote certain physicochemical features correlated with bio-activity.

## Methods

### Sample preparation

After synthesis, the purity of the investigated compounds was confirmed by elemental analysis (C, H, N), chromatography (TLC), and spectral studies (FT-IR/IR, ^1^H NMR, MS)^25^. Next, the spectroscopic pellets were prepared by mixing 10% of a drug in a crystalline form (ground into fine particles in a pestle and mortar) and 90% of high density polyethylene (PE) powder, used as a spectroscopic matrix. The mixture was pressed under 2 tons for 2 minutes, forming 13 mm diameter pellets, which contained 360 mg of the PE powder and 40 mg of the measured substance, whereas the reference tablet was made of 360 mg of pure PE. This methodology should ensure a lack of negative absorbance after background subtraction^30^. There was one exception from the 10% pellet concentration rule – we have introduced a sample, denoted here as 2b, to test the sensitivity of the method to varying the concentration, increased in this case to 20%.

### THz spectroscopy

The pellet samples were measured in a classical terahertz time-domain spectrometer (THz-TDS)^14^. A femtosecond laser with an average power of 200 mW, 85 fs pulse length, and a repetition rate of 100 MHz shone onto the emitting (30 V, 20 kHz square wave bias), and receiving (non-biased) LT-GaAS dipole photoconductive antennas (PCA), which were illuminated by the near-infrared beam (attenuated down to 10 mW) focused using microscopic objectives. The THz pulse from the emitting PCA was guided to the receiver by four off-axis parabolic mirrors in a dry-nitrogen-purged environment. The pulse was reconstructed by moving a retro-reflector mounted on a precession linear stage, and by sampling the signal from the receiving antenna using a lock-in amplifier synchronized with the electronic chopper^31^. A tested compound (pellet) mounted in a rotary sample holder was inserted into the focal point between the second and third parabolic mirror. The usable bandwidth of our system was 2.4 THz, which constrained the upper frequency limit for analysis. We define it here as the spectral region, where the THz signal from a single acquisition is stronger than the system noise by 3 dB. For reliability, after rejection of the featureless low frequency band, the absorbance spectra (obtained using standard mathematical procedures^26^) were analyzed in the range of 20–80 cm^−1^.

### Statistical analysis

In statistical analysis, the THz spectra were first normalized using the Standard Normal Variate (SNV), and then processed using Principal Component Analysis (PCA) and Partial Least Squares (PLS), whereas the molecular descriptors data were subject only to PCA, but were not normalized. A comparison between different normalization techniques considered in our studies together with their impact on the distribution of points on the maps is provided in the Supplementary Information, which additionally lists the molecular descriptors (physicochemical parameters) of the samples in Table S1, and provides a brief mathematical background for the methods used here.

The datasets analysed during the current study are available from the corresponding author on reasonable request.

## Electronic supplementary material

Supplementary information

## Footnotes

1
^†^The crude products were purified by column chromatography using the appropriate eluent.
