# Supplementary material for: Chemometric Evaluation of THz Spectral Similarity for the Selection of Early Drug Candidates
Source: Sci Rep. 2017 Nov 6;7:14583. doi: 10.1038/s41598-017-14819-6 (PMC5674078; doi:10.1038/s41598-017-14819-6)
Supplement: Supplementary file 1 — Supplementary information [file 41598_2017_14819_MOESM1_ESM.pdf]

Supplementary information: Chemometric Evaluation of THz Spectral Similarity for the Selection of Early Drug Candidates

Lukasz A. Sterczewski[1,4,*], Kacper Nowak[1], Boguslaw Szlachetko[1], Michal P. Grzelczak[1], Berenika Szczesniak-Siega[2], Stanislawa Plinska[3], Wieslaw Malinka[2], and Edward F. Plinski[1,§]

[1]Wroclaw University of Science and Technology, Faculty of Electronics, 50-370 Wroclaw, Poland
[2]Wroclaw Medical University, Department of Chemistry of Drugs, 50-556 Wroclaw, Poland
[3]Wroclaw Medical University, Department of Inorganic Chemistry, 50-556 Wroclaw, Poland
[4]Princeton University, Department of Electrical Engineering, Princeton, New Jersey 08544, USA
[*]lukasz.sterczewski@pwr.edu.pl
[§]edward.plinski@pwr.edu.pl

ABSTRACT

This document serves to explain the experimental and numerical procedures used in our studies on the use of THz spectral and molecular descriptors similarity to support the selection of early drug candidates.

Investigated samples

Table S1 provides the list of synthesized and spectroscopically measured Piroxicam analogues including their molecular descriptors. The exact chemical formulas of investigated samples cannot be provided due to a pending patent process[1]. This work, however, is focused on the prediction of selected physicochemical parameters of structurally-similar drugs based on the similarities of THz spectra, therefore providing chemical formulas gives no supplementary information here.

Signal processing

Spectrum preprocessing

In order to obtain the THz spectrum of a Piroxicam derivative, and suppress the instrumental response of the spectrometer we perform deconvolution in the spectral domain:

$$\sqrt{T(\omega)}e^{-i\phi(\omega)} = \frac{\tilde{S}_s(\omega)}{\tilde{S}_r(\omega)} , \tag{S1}$$

where $T(\omega)$ is the power transmittance, $\phi(\omega)$ is the sample-induced phase shift, and $\tilde{S}_s(\omega)$ and $\tilde{S}_r(\omega)$ are the complex frequency spectra registered when a sample or reference pellet is inserted into the THz beam, respectively. The frequency response of each pellet $\tilde{S}_i(\omega)$ is calculated using the discrete Fourier transform of a zero-padded time-domain signal, multiplied by the Hamming window.

$$\tilde{S}_i(\omega) = \sum_{n=0}^{N-1} s_i(n) w_H(n-k) e^{-j\omega n} , \tag{S2}$$

where $s_i(n)$ is a discretely-sampled THz signal registered at the receiving antenna of the spectrometer, $\omega = 2\pi f$ is the angular frequency, and $w_H(n-k)$ is a discrete Hamming window aligned to the center of energy of the THz signal. We calculated the absorbance spectra $A(\omega)$ by taking the negative of the base-10 logarithm of the power transmittance $T(\omega)$. Additionally, the refractive index n from $\phi(\omega)$ and absorption coefficient $\alpha(\omega)$ given the sample thickness can be obtained[2].

Normalization

To ensure an equal contribution of the samples' spectra into the statistical model, the data vectors of length L need to be normalized. Such an operation can suppress the contribution of the spectrally rich samples and amplify the variance of the

Table S1. Drug candidates selected for spectroscopic measurements with their molecular descriptors

N	Sample name	M_r	MP	PSA	HBD/A	log$P$	ROTB
1	1p+3o	503.6	137.0	106.6	1/7	1.71	5
2a	5p+3o (pol.)†	517.6	145.5	106.6	1/7	2.17	5
2b	5p+3o (20%)‡	517.6	145.5	106.6	1/7	2.17	5
66	5p+3o*	517.6	145.5	106.6	1/7	2.17	5
3	6p+3o	521.6	155.5	106.6	1/8	1.85	5
5	1p+1o	503.6	162.5	89.5	1/7	2.32	7
8	4p+10o	563.7	155.5	108.0	1/9	1.81	9
9	5p+10o	547.7	171.5	98.8	1/8	2.53	8
10	1p+22o	513.6	229.5	98.8	2/7	1.05	4
11	5p+1o	517.4	123.0	89.5	1/7	1.81	7
12	6p+1o	521.6	82.5	89.5	1/8	2.46	7
13	1p+11o	521.6	140.5	89.5	1/8	2.46	7
14	5p+11o	535.6	145.5	89.5	1/8	2.93	7
15	6p+11o	539.6	100.0	89.5	1/9	2.60	7
16	1p+10o	533.6	184.0	98.8	1/8	2.07	8
17	1p+4o	533.6	172.5	115.8	1/8	1.45	6
18	1p+7o	504.6	105.0	119.5	1/8	1.09	5
20	5p+2o	585.6	133.5	106.6	1/10	2.17	6
21	8p+2o	650.5	139.5	106.6	1/10	3.38	6
22	8p+8o	584.4	205.0	132.4	1/9	1.04	5
23	1p+9o	571.6	130.0	89.5	1/10	3.20	8
38	2p+3o	538.0	107.0	106.6	1/7	2.22	5
43	4p+3o	533.6	114.0	115.8	1/8	1.45	6
56	8p+3o	582.5	137.5	106.6	1/7	2.50	5
60	1p (no 'o')*	301.3	163.5	91.85	2/5	0.09	2
64	5p (no 'o')*	315.3	231.5	91.85	2/5	0.55	2
65	Piroxicam	331.4	191.5	108.0	2/6	-0.74	2

Note: M_r – molecular mass (g/mol), MP – Melting point (°C, mean of the range), PSA – polar surface area (Å^2), HBD – hydrogen bond donors, HBA – hydrogen bond acceptors, log$P$ – log of octanol-water partition coefficient P evaluated in Accelrys Draw 4.1 (lipophilicity). The number of aromatic rings (AROMs) was equal to 3 for all samples excluding 10th (AROMs = 4), pure Piroxicam (AROMs = 2), 60th (AROMs = 2), and 64th (AROMs = 2).

Note II: (†,‡,*) – samples 2a, 2b, and 66 were structurally identical but their polymorphic forms and concentration were different: (†) was in form I 10%, (‡) was in form II 20%, and (*) was in form II 10%. Sample 2b (‡) was the only exception from the 10% concentration rule. (*) – samples 60 and 64 did not have the structural 'o' group.

weaker ones, thus we have considered a few normalization methods. The normalization to-one (TO) scales the spectrum to the largest peak, whereas the normalization to an area under curve (AUC) scales it by the cumulative sum or trapezoidal integral of the area under the spectral curve. These two methods yield similar results. The third considered method – the Standard Normal Variate (SNV) normalization – removes the mean from the spectra caused by scattering and variation of the particle size[3], therefore it is widely used in Raman, MIR and NIR spectroscopy. Compared to the feature-boosting SNV normalization, the variance of the spectra after the AUC normalization is notably weaker, as shown in Fig. S1. The grouping capabilities of the PCA algorithm applied to the Piroxicam derivatives' spectra normalized with different techniques are shown in Fig. S2. The coordinates of each dot on the spectral similarity map correspond to the PC1 and PC2 scores, whereas the colors represent the groups of substances with a similar cosine distance (obtained with the k-means algorithm).

In statistical analysis we would like to focus on the spectral shape similarities, rather than the absolute value of absorption. This is because, the strong THz absorbers may completely shadow the shape of the weaker ones, thus PCA would not be able to build the proper "spectral mixture" model. For instance, the AUC normalization causes congregation of all the spectra into one group plus a few outliers, therefore this method may be considered unsuitable. In contrast, the TO normalization provides an even distribution of the samples but does not create any visible groups that could be linked to some physical features. Only the SNV normalization gives a discriminative distribution of the results on the PCA map (see Fig. S2) and compensates for the

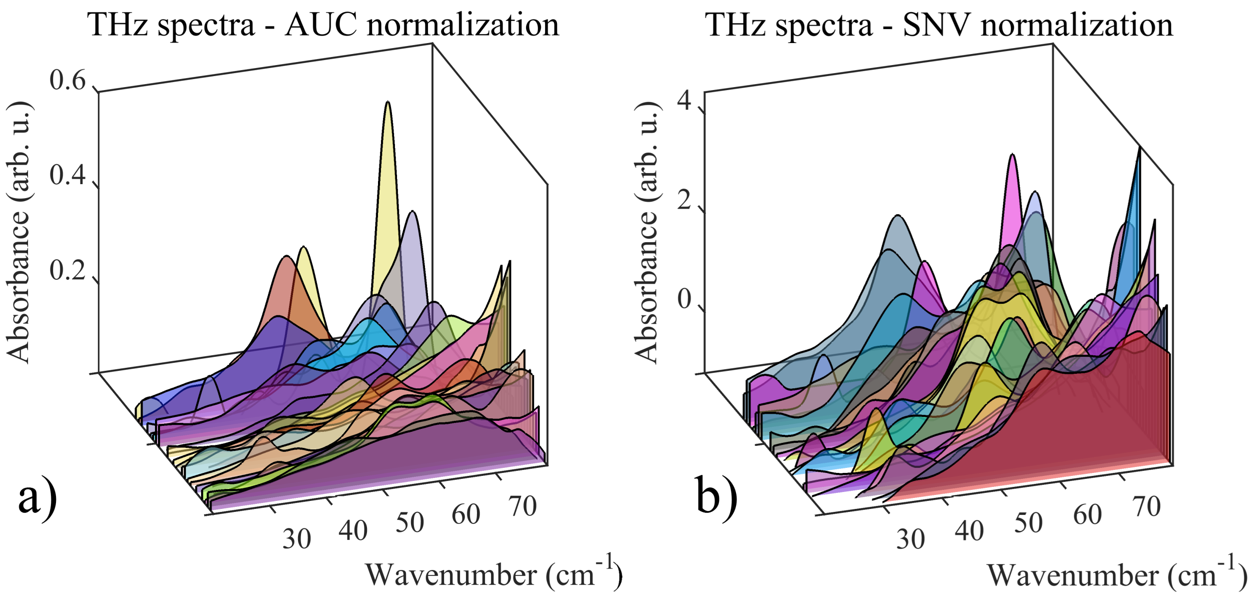

Figure S1. Comparison of variance of Piroxicam analogues spectra for two normalization methods: (a) AUC and (b) SNV. The limits of the ordinate span from the minimum to the maximum of the normalized spectra, which are sorted with respect to their spectral similarity.

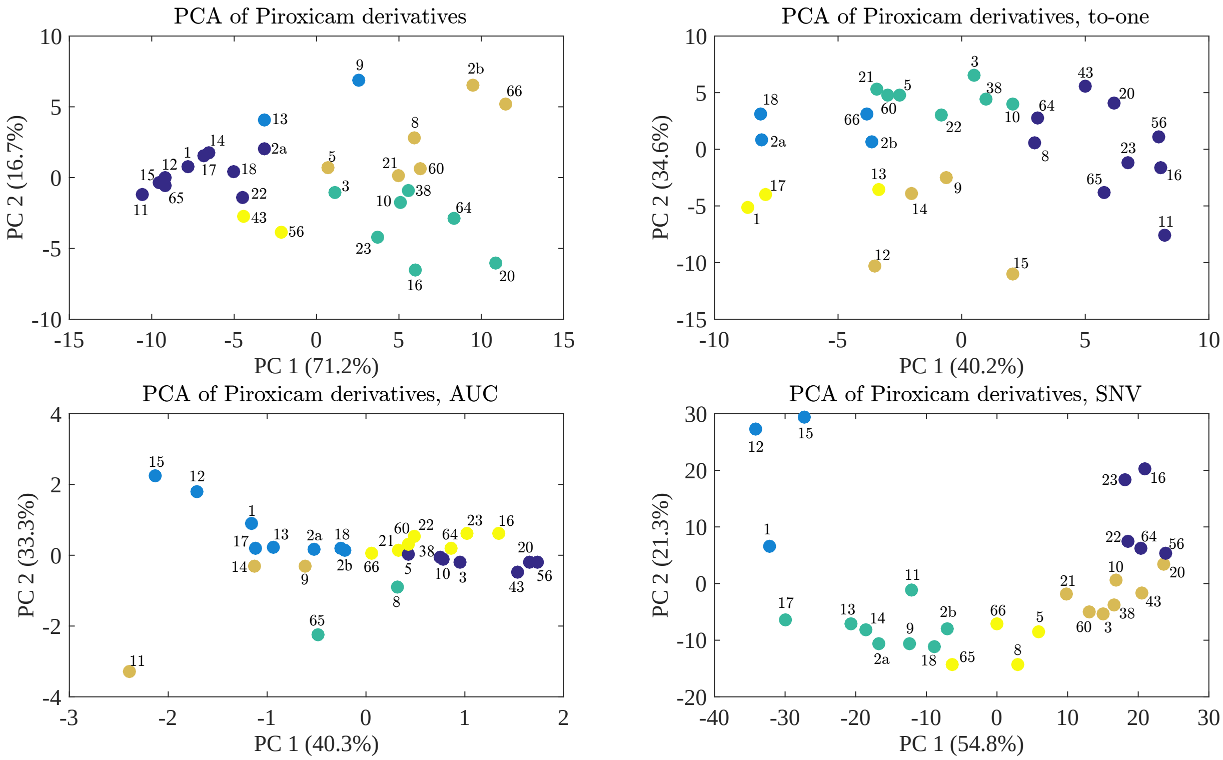

Figure S2. Comparison of grouping capabilities of the PCA method with different spectrum normalization algorithms: none, TO (to-one), AUC, and SNV.

variation of the particle size and scattering effects. In THz spectroscopy, plenty of physical phenomena have a high impact on the results, so choosing the normalization method correlated with underlying physics seems to be crucial. In fact, the SNV normalization technique amplifies the spectral shape of the weaker absorbers, enabling the discovery of a link between the spectral and molecular descriptors domains.

Statistical analysis

For convenience, we provide a brief introduction to Principal Component Analysis and Partial Least Squares regression, which are intensively utilized in our studies.

Principal component analysis

If we denote $\mathbf{s_n}$ as a column vector containing a spectrum or molecular descriptors of a single sample, and subtract the row-wise mean (either the mean of each descriptor or a spectral band), then the correlation matrix $\mathbf{C}$ can be expressed as

$$\mathbf{C} \propto \mathbf{X}\mathbf{X}^\top = [\mathbf{s_1}\ \mathbf{s_2}\ \cdots\ \mathbf{s_n}] \begin{bmatrix} \mathbf{s_1} \\ \mathbf{s_2} \\ \vdots \\ \mathbf{s_n} \end{bmatrix} = \mathbf{W}\mathbf{\Lambda}\mathbf{W}^\top \ . \tag{S3}$$

The covariance matrix $\mathbf{C}$ is factorized by means of eigen-decomposition into two matrices: $\mathbf{W}$, which stores the column eigenvectors $\mathbf{w_i}$, known as the PCA loadings, and $\mathbf{\Lambda}$ storing eigenvalues λ_i on the diagonal. An important feature of the latter matrix is that the eigenvalues appear in descending order with respect to their significance, measured as the variance they explain. Projection of normalized input data $\mathbf{X}$ onto the principal component space (eigenvectors) yields the PCA scores $\mathbf{T}$ of each investigated sample, stored as the column vectors $\mathbf{t_i}$

$$\mathbf{T} = \mathbf{W}^\top\mathbf{X} = [\mathbf{t_1}\ \mathbf{t_2}\ \cdots\ \mathbf{t_n}] \ . \tag{S4}$$

An analysis of the eigenvectors present in $\mathbf{\Lambda}$ reveals that the first few eigenvectors (from even thousands) are usually sufficient to explain most of the data variance. By selecting the first r out of m available (m is the number of variables – molecular descriptors or spectral bands), one can efficiently reduce the dimension of the input problem to a convenient low-dimensional space. For r=2 we reduce the data to two dimensions.

Principal Component Analysis can also be understood in an intuitive way, here supported by a spectroscopic example. If one calculates the weighted average of the ”mixing coefficients” – the PC scores $\mathbf{T}$ (coordinates of the points), and the ”pure spectral sources” – the PC loadings $\mathbf{W}$ (Fig. S3a), one can restore the original data with the compression level depending on the number of eigenvectors taken into account (Fig. S3b). Consequently, PCA assumes that each spectrum is a linear combination of the common ”building spectra” and the ”mixing coefficients”. The PC scores plotted one against the other (as in the parent document) show the similarity of the proportions, in which the different spectra were ”mixed”. As a result, the spatial neighborhood on the PCA map can be understood as the similarity in the original data space – either spectroscopic or molecular descriptor. We have generated such PC score maps in the two stages of our analysis denoted here as *a priori* and *a posteriori*.

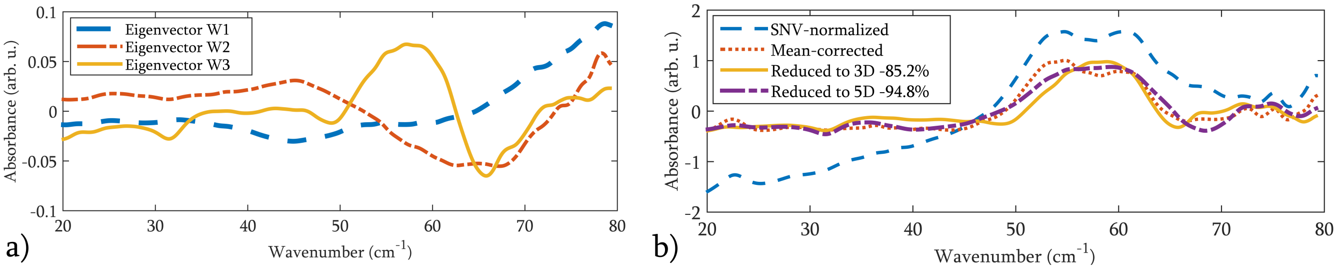

Figure S3. **(a)** Principal component loadings (eigenvectors) and the percentage of variance carried by each of them: $W1$ (54.8%), $W2$ (21.3%), $W3$ (9.1%). **(b)** THz spectra reconstruction of sample 2b from PC scores and loadings: SNV-normalized, row-wise mean corrected (each spectral band), the first three vectors reconstruction, and the first five vectors reconstruction.

Partial least squares

In prediction studies we employed Partial Least Squares (PLS), which is a regression algorithm that uncovers fundamental relations between two matrices of parameters. Conventionally, the PLS method was used to determine the concentration of a substance based on the spectrum[4] utilizing the so-called calibration curve[5]. Since this method well-known in Raman, NIR and MIR spectroscopic studies, and also in on-line chemical process monitoring, we have implemented it in our analysis.

In principle PLS is similar to PCA, but enables to predict some parameters (usually physical) from a given vector of features. In our studies we searched for a relation between the THz spectra of Piroxicam analogues and their physicochemical parameters

(*i.e.* the melting point). The Partial Least Squares method builds a regression model that transforms the input spectra stored in **X** to predict the spectrum-dependent parameters stored in **Y**. Given the regression weights in **B**, by multiplying

$$\mathbf{Y} = \mathbf{XB} + \mathbf{E} \tag{S5}$$

we can estimate an unknown parameter from a THz spectrum with error **E**. In order to build a model **B**, it requires an extensive training, validation and test data set. Due to the lack of these, as the number of our samples was only 27, we introduced a slightly modified strategy to demonstrate the feasibility of such prediction.

A selected number of samples (understood as the THz spectra with parameters from Table S1) are picked in random order from the sample dataset to form two groups: a training and a testing one. Then, the set of the training spectra and the corresponding parameters are used to build the regression model. The testing group is harnessed to evaluate the accuracy of the prediction. In this work, dual, quad and octal groups are selected for evaluation. Consequently the training set was reduced by two, four and eight samples, respectively.

Errors in partial least squares regression

For the sake of evaluation of Partial Least Squares based prediction, let us define the absolute error of prediction of the melting point as follows:

$$e = |T_r - T_p| \,, \tag{S6}$$

where T_r, T_p stand for the real and predicted melting point of a sample in Celsius, respectively. Similarly, the relative error is defined as follows:

$$e_R = \left| \frac{T_r - T_p}{T_r} \right| . \tag{S7}$$

Taking into account that the prediction experiment was run N=5000 times with a different training set in each iteration i, the mean absolute error (MAE) is:

$$\hat{e} = \frac{1}{N} \sum_{i=1}^{N} |T_r - T_p(i)| \,, \tag{S8}$$

and mean relative error (MRE)

$$\hat{e}_R = \frac{1}{N} \sum_{i=1}^{N} \left| \frac{T_r - T_p(i)}{T_r} \right| . \tag{S9}$$

Also, the maximum and minimum value of the error for all prediction experiments can be calculated as follows:

$$e_\alpha = \max_{i \in (1..N)} (T_r - T_p(i)) \,, \tag{S10}$$

$$e_\omega = \min_{i \in (1..N)} (T_r - T_p(i)) \,. \tag{S11}$$

References

1. Szczesniak-Siega, B. *Synthesis and properties of new 1,2-benzothiazine derivatives as potential multitarget drugs with chemopreventive and analgesic activity*. Ph.D. thesis, Faculty of Pharmacy at Wroclaw Medical Academy, Poland (2016).
2. Chen, J., Chen, Y., Zhao, H., Bastiaans, G. J. & Zhang, X.-C. Absorption coefficients of selected explosives and related compounds in the range of 0.1–2.8 thz. *Opt. Express* **15**, 12060–12067 (2007).
3. Barnes, R. J., Dhanoa, M. S. & Lister, S. J. Standard normal variate transformation and de-trending of near-infrared diffuse reflectance spectra. *Appl. Spectrosc.* **43**, 772–777 (1989).
4. Sun, Y. *Comparison and combination of near-infrared and Raman spectra for PLS and NAS quantitation of glucose, urea and lactate*. Ph.D. thesis, University of Iowa (2013).
5. Baek, S. H., Lim, H. B. & Chun, H. S. Detection of Melamine in Foods Using Terahertz Time-Domain Spectroscopy. *J. Agric. Food Chem.* **62**, 5403–5407 (2014). DOI 10.1021/jf501170z.
